# Headaches in adults in supplementary health: management

**DOI:** 10.1590/1806-9282.023D701

**Published:** 2024-03-15

**Authors:** Marcelo Cedrinho Ciciarelli, Caio Vinicius de Meira Grava Simioni, Renata Gomes Londero

**Affiliations:** 1 Universidade Barão de Mauá, Brazilian Academy of Neurology, Faculty of Medicine – São Paulo (SP), Brazil.; 2 Universidade de São Paulo, Brazilian Academy of Neurology, Clinical Hospital, Faculty of Medicine – São Paulo (SP), Brazil.; 3 Brazilian Academy of Neurology, Porto Alegre Clinical Hospital – Porto Alegre (RS), Brazil.

## DESCRIPTION OF THE EVIDENCE COLLECTION METHOD

Research strategy on headache treatment: a search was carried out in PubMed, LILACS, and SciELO with the following search strategy: headache (Mesh Terms) AND treatment (Mesh Terms). With the strategy headache and treatment, or migraine and treatment, the Cochrane secondary database was searched. This initial search, restricted to publications from the past 20 years, resulted in 35,112 articles. Filters were then used for articles published in Portuguese and English, randomized clinical trials (RCTs), and guidelines, resulting in 9782 articles. Excluding articles on the treatment of secondary headaches, cranial neuralgias, and primary headaches other than migraines, tension-type headaches, and cluster headaches, 85 articles were selected for use in preparing this clinical guideline. Inclusion criteria: adult or elderly patients (studies on the pediatric population were excluded), with clinical complaints of headache, with diagnoses of a primary headache compatible with the diagnoses prevalent in the clinic; preferably RCTs, but, in the absence of these for the specific topic, nonrandomized, comparative studies between drugs (not placebo) were included; series and case reports were excluded whenever there was better evidence available; and articles with internal validity and potential external validity for Brazilian reality were included. Exclusion criteria: articles that focus on realities different from the Brazilian one (medicines not available in Brazil); articles in which the internal validity could be questioned; articles aimed at the management of secondary headaches (except for medication overuse headache); and articles whose treatment focus was not medication (manipulation, cognitive behavioral therapy, and others).

## DEGREES OF RECOMMENDATION AND STRENGTH OF THE EVIDENCE

- Experimental or observational studies of better consistency.
- Experimental or observational studies of lower consistency.
- Case reports or case series (uncontrolled studies).
- Opinion devoid of critical assessment, based on consensus, experts, physiological studies, or animal models.

## GOALS:

This study aimed to evaluate the updated what would be the best therapeutic approach for the complaining of headache in adult patients treated in supplementary healthcare (electively), considering the most prevalent diagnoses and the best evidence available to support the approach.

## INTRODUCTION

Headache is the most prevalent neurological condition and the third-most common painful reason for seeking medical care. In all, 50% of the world's population will have at least one headache attack per year, and more than 90% will have one in their lifetime. The average lifetime prevalence of migration is 18%, and the estimated average prevalence during the last year was 13%. Tension-type headache is more common than migraine (lifetime prevalence of approximately 52%), but, as it is less disabling, it less frequently leads the patient to consult.

Tension-type headaches and migraines are frequent causes of absenteeism and presenteeism, with occasional (as a group) being the second-most common cause of years lost due to disability in the world: 7.2 and 44.5 million years lost due to disability in 2015, respectively.

Despite the impressive numbers, it is estimated that more than 70% of people with recurrent headaches in the world do not receive adequate diagnosis and management.

## CLINICAL ISSUES

What is the best treatment to end a current headache attack – symptomatic treatment of the attack?

What is the best treatment to prevent recurrent headaches – prophylactic treatment of different primary headaches?

## GUIDELINES FOR THE ACUTE SYMPTOMATIC MANAGEMENT OF PRIMARY HEADACHES

Symptomatic treatment of primary headaches aims to reduce the intensity or eliminate pain in a sustainable, safe, and accessible way. Correct guidance on the treatment of crises provides functional recovery, avoiding the need for emergency services and reduced work capacity. It is important, however, to raise awareness of the rational use of acute symptomatic medications, as their indiscriminate use can lead to medication overuse headache, a complicating factor in primary headaches^1^.

Staggered x stratified treatment^2^

The choice of acute symptomatic treatment can be made in the following way:

Staggered: treatment begins with the prescription of nonspecific analgesic drugs. At each consultation, the doctor can adjust the symptomatic medication according to the response obtained, taking into account the results of the previous prescription.
Stratified: the doctor, based on the description of the crisis and the patient's previous experience, prescribes a treatment that would be compatible with their intensity and response to treatments already tried.

There is evidence that stratified treatment is more effective in reducing the time for pain relief, the recurrence of attacks, and the need for additional doses of medication, which is why it is recommended in this guideline.

### Acute symptomatic treatment of migraine

The treatment of migraine attacks must be based on individual aspects, since migraine is a complex disease with multiple characteristics that vary from one person to another, which can influence the outcome of treatment. Drugs must be chosen taking into account each patient's history (previous results, allergies, contraindications, and comorbidities)^1^.

Nonspecific and/or specific drugs can be used^3^. Specific drugs are triptans and ergot derivatives. Nonspecific drugs are simple analgesics and nonhormonal anti-inflammatory drugs (NSAIDs). The combined use of antiemetics, neuroleptics, and corticosteroids may be necessary. Opioids, however, should be avoided^4^. For doses, route of administration, and grade of recommendation for the use of different medications, see Tables 1 and 2. For the main studies that supported the recommendation, see Table 3.

**Table 1:** Medications used in migraine attacks: dose, route of administration, and grade of recommendation.

Medication	Dose	Route of administration	Grade of recommendation
Paracetamol	1000 mg	PO	A
Dipyrone	1000 mg	PO	B
Naproxen	500/550 mg	PO	A
Ibuprofen	200/400 mg	PO	A
Diclofenac	50/100 mg	PO	A
Acetylsalicylic acid	500 mg	PO	A
Naratriptan	2.5 mg	PO	A
Rizatriptan	5 mg	PO	A
Sumatriptan	25/50/100 mg	PO	A
	10 mg	NASAL	
	6 mg	SC	
Zolmitriptan	2.5 mg	PO	A
Sumatriptan/naproxen	85/500 mg	PO	A
Paracetamol/acetylsalicylic acid/caffeine	500/500/300 mg	PO	A
Chlorpromazine	12.5 mg	IM	B
Metoclopramide	10 mg	IV	B
Ketoprofen	100 mg	PO	B
Ketorolac	30/60 mg	IV/IM	B
Magnesium sulfate (migraine with aura)	1–2 g	IV	B
Dexamethasone	4–16 mg	IV	C

**Table 2:** Medications used to treat migraine attacks: reviewed studies and grade of recommendation.

Medication	Author, year (n)	Result	Grade of recommendation
Acetaminophen	Freitag, 2008 (173)^3^, Prior, 2010 (346)^17^	Acetaminophen superior to placebo	A
Dipyrone	Bigal, 2001 (269)^7^; Bigal, 2002 (74)^18^	Dipyrone superior to placebo	B
Acetylsalicylic acid	Lipton, 2005 (485)^19^, MacGregor, 2002 (101)^20^	Acetylsalicylic acid superior to placebo	A
Ibuprofen	Codispoti, 2001 (660)^21^, Diener, 2004 (312)^22^; Misra, 2007 (124)^23^	Comparable to sumatriptan and acetylsalicylic acid+metoclopramide; superior to placebo, inferior to zolmitriptan	A
Naproxen	Nestvold, 1985 (41)^24^, Johnson, 1985 (70)^25^; Wentz, 2008 (337)^26^, Smith, 2005 (972)^27^	Superior to placebo	A
Sumatriptan	Smith, 2005 (972)^27^; Bussone, 2000 (233)^28^	Superior to placebo	A
Sumatriptan + naproxen	Smith, 2005 (972)^27^	Superior to sumatriptan alone, naproxen alone, placebo	A
Rizatriptan	Freitag, 2008 (173)^3^, Seeburger, 2011 (102)^29^	Superior to placebo and paracetamol; superior to placebo in nonresponders to sumatriptan	A
Zolmitriptan	Misra, 2007 (124)^23^	Superior to ibuprofen and placebo	A

**Table 3:** Medicines, nutraceuticals, and devices used in the prophylaxis of episodic migraine, doses, indications, and side effects.

Medication	Starting dose	Maintenance dose	Additional beneficial effects	Side effects	Grade of recommendation
Propranolol^36^	10 mg BID	80–240 mg, BID or TID	Essential tremor, heart rate control, antihypertensive	Tiredness, asthma exacerbation, decreased libido, depression, increased triglycerides	A
Metoprolol succinate^37^	25 mg qd	100–200 mg qd	Heart rate control, antihypertensive	Decreased libido, depression, increased triglycerides Better tolerated than propranolol	A
Topiramate^38,39^	25 mg at night	25–100 mg at night or BID 25 mg increase every 4 weeks	Reduction of body weight, indicated for headache secondary to idiopathic intracranial hypertension, mood stabilizer, anti-epileptic	Contraindicated during pregnancy; interacts with contraceptives, which may reduce their effectiveness	A
Valproato^40^	250 mg 12/12 hours	500–1500 mg BID	Mood stabilizer, anti-epileptic	Contraindicated during pregnancy, avoid in women at risk of pregnancy, weight gain, hair loss	A
Divalproato^41,42^	250 mg qd	250–1500 mg qd	Mood stabilizer, anti-epileptic	Contraindicated during pregnancy, avoid in women at risk of pregnancy	A
Atenolol^43^	25 mg qd	50–200 mg qd	Heart rate control, greater antihypertensive effect	Decreased libido, depression, increased triglycerides Better tolerated than propranolol	B
Amitriptilina^38^	10 mg at night	10–200 mg at night	Improves sleep, antidepressant, in comorbidity with tension-type headache	Constipation, dry mucous membranes, palpitation	B
Nortriptilina* ^44^	10 mg at night	10–200 mg at night	Antidepressant, in comorbidity with tension-type headache	Constipation, dry mucous membranes, palpitation (less common than with amitriptyline)	–
Venlafaxine^45,46^	37.5 mg qd	75–225 mg qd	Management of depression, anxiety, changes in sleep.	Weight loss, nausea, vomiting.	B
Candesartan^47^	8 mg in the morning	8–16 mg daily	Antihypertensive Indicated as an adjuvant	Contraindicated during pregnancy	C
Lisinopril^48^	5 mg in the morning	5–10 mg in the morning	Antihypertensive Indicated as an adjuvant	Contraindicated during pregnancy	C
Gabapentina^49^	300 mg at night	300–1800 mg 12/12 hours	–	–	U
Verapamil^50^	40 mg BID	180–480 mg BID	Heart rate control	Lower limb edema	U
Flunarizine^36,51^	5 mg at night	5–10 mg at night	Anti-vertigo effect, improves sleep	Weight gain, drowsiness, parkinsonism with prolonged use	–
Eptinezumab*** ^52^	100 mg IV	100–300 mg IV	–	High cost, need for application in hospital environment	A
Erenumab** ^53,54^	70 mg	70–140 mg/month SC	–	High cost	A
Fremanezumab^55,56^	225 mg/month SC 675 mg/3 months SC	225 mg/month SC 675 mg/3 months SC	–	High cost	A
Galcanezumab^57^	240 mg/month - 1^a^ dose	120 mg/month SC	–	High cost	A
Magnesium^58^	400–600 mg qd	400–600 mg qd	Safe during pregnancy and breastfeeding Has an effect on constipation	Diarrhea	B
Coenzyme Q-10^59,60^	300 mg qd	300 mg qd	Safe during pregnancy and breastfeeding	–	C
Riboflavin^60,61^	400 mg qd or 200 mg BID	400 mg qd or 200 mg BID	No side effects	Because it is used at a dose above the physiological level, the safety of use during pregnancy is still under discussion	B
Electrical stimulation of the supraorbital nerve^62^	Specific protocol	20 min, once a day	Safe during pregnancy and breastfeeding	Discomfort at the site: from paresthesia to a slight sensation of shock	B

qd: single dose; Grade A evidence: established efficacy; grade B: probable efficacy; grade C: possible efficacy; grade U: inadequate data or conflicting evidence, (–) with no degree of evidence defined to date.

* Medication widely used when amitriptyline causes drowsiness, but without studies proving effectiveness.

** At the time of publication, approved for use by ANVISA but no longer available for sale.

*** At the time of publication, approved for use by ANVISA but not available for commercialization.

#### Acute symptomatic treatment of migraine during pregnancy/lactation

Pregnancy can cause changes in the previous pattern of migraine. A reduction in the frequency and intensity of attacks, as well as a faster response to symptomatic medications, usually occur during pregnancy. Less frequently, the remission, worsening, or even onset of migraine attacks for the first time may be observed.

This treatment guideline emphasizes pharmacological measures and their respective levels of evidence; however, it is important to highlight that during pregnancy and lactation, preference is given to nonpharmacological measures, particularly for less intense painful episodes. If there is a need for drug treatment, it is always necessary to evaluate the relationship between risk and benefit for the fetus. However, the weak scientific evidence related to maternal-fetal efficacy and safety must be taken into account.

### Acute symptomatic treatment of tension-type headache

Most tension-type headache attacks are mild to moderate in intensity, so patients often self-medicate with simple analgesics (e.g., paracetamol or acetylsalicylic acid) or NSAIDs. The effectiveness of simple analgesics tends to decrease with increasing headache frequency.

Even so, simple analgesics^5^ and NSAIDs^6^ are the main treatments for tension-type headache attacks. Paracetamol is less effective than NSAIDs but has fewer gastric adverse effects. Combinations with caffeine-containing analgesics are more effective than simple analgesics and NSAIDs^6^; however, they increase the risk of headaches due to excessive medication use. Triptans, myorelaxants, and opioids are not indicated for the acute symptomatic treatment of tension-type headaches. Medications, doses, and grade of recommendation are presented in Table 4.

**Table 4:** Oral medications used to manage tension-type headache attacks: dose and grade of recommendation.

Medication	Dose (PO)	Grade of recommendation
Dipyrone (Metamizol)^5,7^	500–1000 mg	A
Ibuprofen^8,9^	200–400 mg	A
Ketoprofen^9^	25–50 mg	A
Acetylsalicylic acid^5^	500–1000 mg	A
Naproxen^9^	375–550 mg	A
Diclofenac^10^	12.5–100 mg	A
Paracetamol^11^	1000 mg	A
Combinations with caffeine^6,12^	65–200 mg	B

### Acute symptomatic treatment of cluster headache

Cluster headache attacks are considered the most serious among primary headaches due to their very intense intensity, association with autonomic symptoms, and high daily frequency. Furthermore, a reasonable proportion of patients with cluster headaches have the chronic form of the disease, characterized by short periods or lack of remission. Subcutaneous sumatriptan^13^ and mask oxygen inhalation^14,15^ remain at recommendation grade A. The form of prescription for these, recommendation grade, and other drugs also prescribed for the condition are presented in Table 5.

**Table 5:** Medications used in cluster headache attacks: dose, route of administration, and grade of recommendation.

Medication	Dose	Route of administration	Grade of recommendation
Sumatriptan	6 mg	SC	A
Oxygen	100% 6–12 l/min	Nasal (mask)	A
Sumatriptan	10 mg	Nasal (spray)	B
Zolmitriptan	5–10 mg	PO	B
Lidocaine	10%	Nasal (spray)	C

The transitional treatment with the best recommendation grade, B, consists of anesthetic block of occipital nerves with corticosteroids^16^.

## GUIDELINES FOR THE PROPHYLACTIC MANAGEMENT OF PRIMARY HEADACHES

### Episodic migraine prophylaxis

Defining episodic migraine: it is characterized by migraine that occurs between 3 and 14 days per month in the last 3 months.

For those who prescribe prophylactic therapy: Any patient with migraine who presents with headache 4 or more days per month or 8 or more days with headache in the last 3 months is a candidate for prophylactic treatment. Beta blockers, tricyclic and dual antidepressants, and anticonvulsants are usually used. Additionally, there are non-drug methods indicated for prophylaxis: acupuncture^30,31^, biofeedback^32^, cognitive-behavioral therapy, aerobic exercises, and electrical stimulation (transcutaneous electrical stimulation of the supraorbital nerve). These can be adopted in association with drug prophylaxis or as isolated therapy, in this case, especially for pregnant women, breastfeeding women, people who prefer non-drug methods, or who are intolerant of available medications.

Expected benefits for the patient who receives prophylactic treatment are as follows: (1) reduction in the number of days with pain, (2) reduction in pain intensity, (3) reduction in the duration of attacks, and (4) improvement in the response to medications used for relief of attacks (symptomatic medications). Furthermore, evidence suggests that the use of prophylactic medication can prevent the progression of migraine.

General principles of migraine prophylaxis (see Table 6), adapted from Dodic (2018)^33^: the drug is chosen taking into account comorbidities, associated diseases, medications previously used by the patient, and a pregnancy plan. Medications are usually started at a low dose, with a progressive increase after subsequent reassessments, which improves tolerance to the potential side effects of medications. Use for a minimum period of 2–3 months is necessary to assess effectiveness^34^. Several groups of medications have already been tested for prophylactic use, including beta blockers, tricyclic and dual antidepressants, neuromodulators, anticonvulsants, and CGRP inhibitors. See Table 3 for drugs, starting and maintenance doses, potential associated beneficial effects, and most prevalent evidence-based paraeffects^35^.

**Table 6:** General principles of migraine prophylaxis.

1. Start with medication at a low dose and increase slowly – usually every 2 weeks, at least.
2. Use the medication for at least 2–3 months, except in the event of intolerable side effects.
3. Pay attention to contraindications and drug interactions.
4. Reinforce the use of the headache diary as a way of monitoring treatment.
5. Watch out for excessive use of painkillers.
6. Assess possible comorbid conditions that aggravate migraine.
7. Consider a combination of prophylactic agents from different categories for refractory patients.
8. Reduce and withdraw prophylaxis when the crises are controlled, in general for 3 months with less than 3 days of pain per month.

### Tension-type headache prophylaxis

Tension-type headache is very common, with a prevalence in the general population varying between 30 and 78% in different studies. Although already considered primarily psychogenic, several studies suggest a neurobiological basis for at least the most severe subtypes of tension-type headache. Tension type headache is divided into episodic and chronic types (more than 15 days per month). The episodic form was subdivided into an infrequent type (less than 12 days per year with pain) and a frequent type (12 or more to less than 180 days per year with pain). Frequent episodic tension-type headaches can be associated with considerable disability and require treatment with medications. Chronic tension-type headache is a serious illness that causes a major decline in quality of life and a high degree of disability and must invariably be managed with prophylaxis. For commonly prescribed drugs, doses, and degrees of recommendation, see Table 7.

**Table 7:** Prophylactic treatment of tension-type headache.

Drug	Dose	Grade of recommendation
Amitriptyline^64,65^	25–75 mg PO	A
Mirtazapine^66^	30 mg PO	B
Venlafaxine^45,67^	150 mg PO	B
Clomipramine^68^	75–150 mg PO	B
Maprotiline^69^	75 mg PO	B
Mianserin^68^	30–60 mg PO	B

Diagnostic criteria for episodic tension-type headache ICHD-3:^63^

- Lasting from 30 min to 7 days
- At least two of the following four characteristics
  1. Bilateral location
  2. Pressing or tightening (nonpulsating) quality
  3. Mild or moderate intensity
  4. Not aggravated by routine physical activity such as walking or climbing stairs
- Both of the following:
  1. No nausea or vomiting
  2. No more than one of photophobia or phonophobia

Diagnostic criteria for chronic tension-type headache:^1^

- Headache occurring on ≥15 days/month on average for >3 months (≥180 days/year), fulfilling criteria B-D
- Lasting hours to days, or unremitting
- At least two of the following four characteristics:
  1. Bilateral location
  2. Pressing or tightening (nonpulsating) quality
  3. Mild or moderate intensity
  4. Not aggravated by routine physical activity such as walking or climbing stairs

Both of the following:

No more than one of photophobia, phonophobia, or mild nausea

Neither moderate or severe nausea nor vomiting

Not better accounted for by another ICHD-3 diagnosis.

### Cluster headache prophylaxis

Defining cluster headache: cluster headache is characterized by symptoms that recur in short periods, one to eight times a day, daily, for a few weeks or months. It is characterized by sudden and intense, fixed unilateral, ocular, or periorbital pain, associated with at least one of the following: conjunctival injection and/or tearing; nasal congestion and/or rhinorrhea; eyelid edema; frontal and facial sweating; miosis and/or ptosis; a feeling of restlessness or agitation.

For which patient to prescribe prophylactic therapy: for every patient with cluster headache.

Expected benefits for the patient who receives prophylactic treatment are as follows: (1) reduction in days with pain, (2) reduction in pain intensity, (3) reduction in attack duration, and (4) improvement in the response to medications for relief of crises (symptomatic medications).

General principles of cluster headache prophylaxis (see Table 8): Cluster headache management includes the use of acute medications for the attack (see a specific chapter on acute management), prophylactic treatment, and transitional treatment. Transitional treatment consists of prescribing medications that take effect faster than prophylactic ones but must be used for a short period of time. It is indicated in two situations: (1) as isolated prevention for patients with short cycles of pain and (2) as a "bridge" for patients with long cycles of pain while another preventive medication is adjusted. The main treatments included here are occipital nerve block (using local anesthetics associated with corticosteroids) and a course of oral corticosteroids. Blocking is usually carried out once and can be repeated within a minimum period of 3 months. The course of oral corticosteroids should last a maximum of 3 weeks and should not be repeated more than two to three times a year. Both time limitations mentioned are due to the side effects of frequent use of corticosteroids. The prophylactic treatment with the best established efficacy is verapamil. If this fails or in cases where it is contraindicated or not tolerated, the options are: topiramate and lithium, and, with less evidence (case series and expert opinions), sodium valproate, baclofen, and testosterone replacement therapy. Melatonin may also be indicated, usually as an adjunct treatment. For refractory cases, sphenopalatine ganglion block and occipital nerve stimulation are still available. Prophylaxis should be started as soon as the diagnosis is established, and slow reduction and subsequent suspension can be considered after the patient remains asymptomatic for at least two weeks.

**Table 8:** Medications and procedures used in cluster headache prophylaxis, doses, and effects.

Medication/procedure	Starting dose	Maintenance dose	Side effects	Grade of recommendation
Verapamil^70^	80 mg BID	80–320 mg, TID	Prolongation of the T interval, tremor	A
Galcanezumab^71^	300 mg SC monthly	300 mg SC monthly	Rare: constipation	A
Oral corticosteroid^72^	Prednisone/prednisolone 1 mg/kg, in the morning	Slow reduction over 2–3 weeks	Hip osteonecrosis, lack of blood pressure and glycemic control	A
Ipsilateral greater occipital nerve block^73,74^	Lidocaine 1–2% 1–4 mL, or bupivacaine 0.25–0.5%, 1–4 mL Associated with 80 mg methylprednisolone	Lidocaine 1–2% 1–4 mL, or bupivacaine 0.25–0.5%, 1–4 mL Associated with 80 mg methylprednisolone	Pain at the application site, rare: hair loss at the application site	B
Lithium^75^	300 mg at night	900 mg/day, serum level 0.7–1.2 mmol/L	Polyuria	B
Topiramate^76^	50 mg at night	100–400 mg qd	Paresthesia, drowsiness, changes in mood, taste	B
Melatonin^77^	10 mg at night	10 mg at night	No reported adverse effects	C
Clomiphene^78,79^	300 mg for 3 days	50 mg for 45–180 days	Acne, ovarian cyst	C

### Prophylaxis of chronic migraine associated or not with headache due to excessive use of analgesics

The current International Classification of Headaches^63^ (ICHD-3) sets up a specific chapter for chronic migraine (CM) and characterizes it as pain that occurs more than 15 days a month for a period longer than 3 months without excessive use of symptomatic medications; as long as at least 8 days of the month, the pain presents typical characteristics of a migraine crisis. During the anamnesis, it is important to highlight the previous history of episodic migraine and its evolutionary nature, which is often associated with the loss of migraine characteristics (see Diagnostic criteria for chronic migraine ICHD-3 in https://ichd-3.org/1-migraine/1-3-chronic-migraine/). Chapter 8 of ICHD-3 covers pre-existing headache, which, in association with excessive use of analgesics, causes a significant worsening of pain frequency. This is characterized by a headache that occurs 15 or more days per month, and its progression was a consequence of the excessive and regular use of symptomatic medications (10 or more days with symptomatic medication, 15 or more days with symptomatic medication, depending on the medication) for a period longer than 3 months. The headache usually improves when use is stopped (see Criteria for headache attributed to medication overuse, according to ICHD-3^63^, in https://ichd-3.org/8-headache-attributed-to-a-substance-or-its-withdrawal/8-2-medication-overuse-headache-moh/).

The treatment of CM should always be preceded by a careful review of the diagnosis, detection of possible worsening factors and associated conditions, stratification of severity/intractability, and monitoring with a pain diary.

Regarding therapeutic measures for CM, prophylactic treatment should always be prioritized over acute treatment. If severe and disabling crises occur, analgesia should be stimulated by nonpharmacological methods.

Prophylactic pharmacological management of CM (Table 9) is always indicated. The association of CM with medication-overuse headache may require, although in a minority of cases, management in hospital. The criteria for defining this need are described in Table 10. Removing excessively used medications can be very challenging, and transitional treatment can be of great value in this case (Table 11).

**Table 9:** Prophylactic treatment of chronic migraine.

Drug	Dose	Grade of recommendation
Onabotulinum toxin type A^80,81^	155–195 UI/cycle, repeated every 12 weeks, for at least the 2–3 cycles	A
Topiramate^38,39,82^	50–100 mg BID PO	A
Divalproex^83^	1000 mg/day PO	B
Amitriptyline^38^	10–200 mg/day PO	A
Galcanezumab^84^	120 mg/month SC	A
Fremanezumab^56,85^	225 mg/month or 675 3/3 months SC	A

**Table 10:** Situations to consider initial management of migraine in an inpatient setting.

Lack of response to appropriate treatment on an outpatient basis.
History of frequent visits to emergency units.
Migraine status or crisis refractory to acute treatment in the emergency unit.
Intense nausea, vomiting, or diarrhea causing dehydration, water and electrolyte disturbance, and/or preventing oral treatment. Special attention should be paid to conditions such as pregnancy, postpartum period, chronic renal failure, severe ischemic heart disease, and arrhythmias.
Changes in vital hemodynamic (blood pressure and heart rate) and respiratory (respiratory rate and O_2_ saturation) data.
Need to stop the excessive use of symptomatic medications (acute analgesics and antimigraine drugs) and the treatment of manifestations related to toxicity and/or dependency/rebound phenomena that cannot be safely managed on an outpatient basis (parenteral treatment and/or intensive symptom monitoring).
Subentrant epileptic seizures or status epilepticus, severe allergic reactions, renal or hepatic failure, thrombocytopenia, bleeding, vascular insufficiency, and serious infection.
Concomitant need for psychiatric hospitalization (risk of aggression, suicide, moral exposure, severe psychosis, detoxification of drug addicts, and abstinence).
When reviewing the diagnosis, it requires procedures best performed in a hospital setting.
Presence of psychosocial factors that prevent adequate treatment outside a controlled environment.

**Table 11:** Transitional treatment of chronic migraine associated with headache due to excessive use of analgesics.

Discontinuation of the drug in excessive use	Treatment of rebound headache	Treatment of withdrawal symptoms
Abrupt in the case of analgesics	Try nonpharmacological measures	Antiemetics
Gradual in cases of excessive use of barbiturates, benzodiazepines, and opioids	Use of unused analgesics, limited to twice a week	Corticosteroids for 7–14 days

Diagnostic criteria for chronic migraine ICHD-3^1^

- Headache (migraine-like or tension-type-like) on ≥15 days/month for >3 months, and fulfilling criteria B and C.
- Occurring in a patient who has had at least five attacks fulfilling criteria B–D for 1.1 *Migraine without aura* and/or criteria B and C for 1.2 *Migraine with aura*.
- On ≥8 days/month for >3 months, fulfilling any of the following:
  1. Criteria C and D for 1.1 *Migraine without aura*
  2. Criteria B and C for 1.2 *Migraine with aura*
  3. Believed by the patient to be migraine at onset and relieved by a triptan or ergot derivative
- Not better accounted for by another ICHD-3 diagnosis.

Diagnostic criteria for medication overuse headache, according to ICHD-3^1^

- Headache occurring on ≥15 days/month in a patient with a pre-existing headache disorder.
- Regular overuse for >3 months of one or more drugs that can be taken for acute and/or symptomatic treatment of headache.^1,2,3^
- Not better accounted for by another ICHD-3 diagnosis.

## Footnotes

1 Patients should be coded for one or more subtypes of 8.2 Medication-overuse headache according to the specific medication(s) overused and the criteria for each below. For example, a patient who fulfills the criteria for 8.2.2 *Triptan-overuse headache* and the criteria for one of the subforms of 8.2.3 *Non-opioid analgesic-overuse headache* should receive both of these codes. The exception occurs when patients overuse combination-analgesic medications, who are coded 8.2.5 *Combination-analgesic-overuse headache and not according to each constituent of the combination-analgesic medication*.

2 Patients who use multiple drugs for acute or symptomatic treatment of headache may do so in a manner that constitutes overuse even though no individual drug or class of drug is overused; such patients should be coded 8.2.6 *Medication-overuse headache attributed to multiple drug classes not individually overused*.

3 Patients who are clearly overusing multiple drugs for acute or symptomatic treatment of headache but cannot give an adequate account of their names and/or quantities are coded 8.2.7 *Medication-overuse headache attributed to unspecified or unverified overuse of multiple drug classes* until better information is available. In almost all cases, this necessitates diary follow-up.
